# DNA Repair Genes Are Associated with Subtype Classification, Prognosis, and Immune Infiltration in Uveal Melanoma

**DOI:** 10.1155/2022/1965451

**Published:** 2022-01-19

**Authors:** Tao Wang, Dingwei Liu, Lin Wang, Mengfan Liu, Wenwen Duan, Jinlin Yi, Yunmin Yi

**Affiliations:** ^1^Affiliated Eye Hospital of Nanchang University, Nanchang 330006, China; ^2^Department of Gastroenterology, The First Affiliated Hospital of Nanchang University, Nanchang 330006, China

## Abstract

Uveal melanoma (UM) is the most common primary intraocular malignancy in adults. DNA repair genes play a vital role in cancer development. However, there has been very little research about DNA repair genes in UM. This study aimed to evaluate the importance of DNA repair genes and established a signature for predicting prognosis and immune features of UM. In this study, we mined TCGA database through bioinformatics analysis, and the intersect was taken between DNA repair genes and prognosis related genes and yielded 52 genes. We divided 80 UM patients into C1 and C2 subtypes. GSEA results indicated that abundant cancer-promoting functions and signaling pathways were activated in C2 subtype and the proportion of SNVs was higher in C2 than in C1 which suggested a worse prognosis. We built a six DNA repair genes model including ITPA, CETN2, CCNO, POLR2J, POLD1, and POLA1 by LASSO regression to predict prognosis of UM patients and utilized the median value of risk scores as the cutoff point to differentiate high risk and low risk group. The survival analyses and the receiver operating characteristic (ROC) curves in the validation group and entire data set confirmed the accuracy of this model. We also constructed a nomogram based on age and risk scores to evaluate the relationship between risk scores and clinical outcome. The calibration curve of the overall survival (OS) indicated that the performance of this model is steady and robust. Finally, the enrichment analysis showed that there were complex regulatory mechanisms in UM patients. The immune infiltration analysis indicated that the immune infiltration in C2 in the high risk group was different from that in the low risk group. Our findings indicated that the DNA repair genes may be related to UM prognosis and provide new insight into the underlying mechanisms.

## 1. Introduction

Uveal melanoma (UM), which arises from melanocytes of the choroid (90%), ciliary body (6%), or iris (4%) of the eye, is the most common primary intraocular malignancy in adults [1,2]. Seven thousand people worldwide are diagnosed with UM each year [3]. The incidence of UM in the United States (5.1 per million per year) has remained unchanged from 1973 to 2008 [4]. The incidence of UM is low in Africa (0.3 per million per year) and Asia such as Japan (0.6 per million per year) [5, 6]. In the past 30 years, although the success of UM with surgery or radiotherapy is high, more than 50% of UM patients still have systemic metastasis [7]. During 3-, 5-, and 10-year follow-up, Carol found that the metastasis rates of more than 8000 UM patients were 8%, 15%, and 25%, respectively [8].There is no effective treatment strategy for metastatic UM patients, whose median survival time is less than 1 year [9,10]. Several pathological factors are related to tumor metastasis and prognosis such as cell type, large size, intrascleral and extrascleral invasion, ciliary body involvement, and optic nerve extension [11–13].

DNA damage can lead to gene mutation, contributing to cancer and other related diseases [14]. DNA repair process, including base excision repair, nucleotide excision repair, mismatch excision, and homologous recombination, is constantly active when DNA damage occurs [15]. Abnormal DNA repair, considered as a hallmark of cancer cells, often occurs in the evolution of many malignancies [16]. Research in the field of UM has mainly focused on genetic, with the aim of unravel UM biology, and revealed potential therapeutic targets [17,18].

However, it is still unclear whether and how DNA repair genes are involved in the occurrence and development of UM. Therefore, it is of clinical significance to find genes that are associated with prognosis in UM based on DNA repair gene expression profiles. Some studies have explored the relationship between gene sets and disease through bioinformatics analysis and established risk prediction models [19, 20]. In our study, we divided two subtypes on the basis of DNA repair genes for UM and a risk score model to evaluate prognosis.

## 2. Materials and Methods

The transcriptome data and simple nucleotide variant (SNV) of 80 UM patients were downloaded from The Cancer Genome Atlas database (TCGA, https://cancergenome.nih.gov/). The relevant clinicopathological characteristics including age, gender, and tumor stage were downloaded from the UCSC Xena website (https://xena.ucsc.edu/). The detailed clinical characteristics are shown in Table 1.

### 2.1. Identification of DNA Repair Gene-Based Subtypes

Univariate cox regression analysis was performed on all genes, and the prognosis-related genes were identified with criteria of *p* < 0.05.150 DNA repair genes were extracted from the gene set, “HALLMARK_DNA_REPAIR,” from Molecular Signatures Database v7.4. Then, we obtained 52 DNA-repair genes, which had statistically significant hazard ratio (HR) related to patient overall survival (OS). Finally, the expression level of intersecting genes were used to identify new subtypes using NMF *R* package and evaluated the best *k* value [21]. Then, we used the principal component analysis (PCA) to evaluate whether the subtype classification is reasonable. Next, the survival curve was used to evaluate the difference in prognosis of different subtypes. We randomly selected 8 genes to further compare the differences of expression levels in the subtypes.

### 2.2. SNVs in the Subtypes

We used tumor mutation data obtained from TCGA to analyze the SNVs in the subtypes and calculated the mutation frequency of all genes using maftools package [22]. The top 30 genes with the most significant mutation frequency difference were listed by waterfall plots.

### 2.3. Enrichment Analysis in the Subtypes

GSEA were performed using the clusterProfiler R package in different subtypes to clarify the key processes activated in subtypes with poor prognosis [23]. We also analyzed the hallmark gene sets to deduce their functions and determined whether the gene sets differed significantly between the subtypes. The research was to explore the differences in functions and pathways between the subtypes.

### 2.4. Development of a Risk Prediction Model Based on DNA Repair Genes

80 UM patients from TCGA were randomly divided into a training group (*n* = 56) and an internal validation group (*n* = 24). In the training group, we selected 6 genes out of 52 DNA repair genes to construct a risk model through lasso regression. Patients were divided into the high risk group and low risk group by the median risk score. Then, we evaluated the risk model in the training group by survival analysis and 1-, 2-, and 3-year ROC curve. At the same time, we conducted validation in the internal validation group and the entire data set to validate the predictive effect of the model.

### 2.5. Establishment and Evaluation of Clinical Prognostic Model

We performed Univariate and multivariate Cox regression analysis on clinicopathological parameters and risk scores including age, gender, and tumor stage to analyze their predictive ability for OS. A nomogram was constructed based on age and risk scores. The 1-, 2-, and 3-year survival time of each patient was predicted according to the nomogram. Meanwhile, the AUC (area under the curve) and calibration curve were used to evaluate the prediction efficiency of the model. The analysis of this study is in accordance with the TRIPOD statement (http://www.tripod-statement.org/).

### 2.6. Functional Enrichment Analysis

The Gene Ontology (GO) enrichment analysis and Kyoto Encyclopedia of Genes and Genomes (KEGG) pathway analysis were applied to differentially expressed genes between the high risk and low risk groups. *P* < 0.05 was considered statistically significant.

### 2.7. Immune Cell Infiltration Levels between the High Risk and Low Risk Subgroups of UM Patients

In order to compare the proportion of different immune cells in the group with the worst prognosis and the low risk group, we analyzed 22 human immune cell phenotypes in the tumor immune microenvironment (TME) using CIBERSORT algorithm. Mann–Whitney *U*-test was used to compare the infiltration level of immune cells between the two groups.

### 2.8. Statistical Analysis

All statistical analyses were performed using R version 4.0.5. Cox regression was used to identify prognosis-related genes, setting *P* < 0.05 as the cutoff value for significance. In all other analyses, *P* < 0.05 was the threshold for statistical significance.

## 3. Results

### 3.1. Selection of Prognostic DNA Repair-Related Regulators

**Figure 1:**
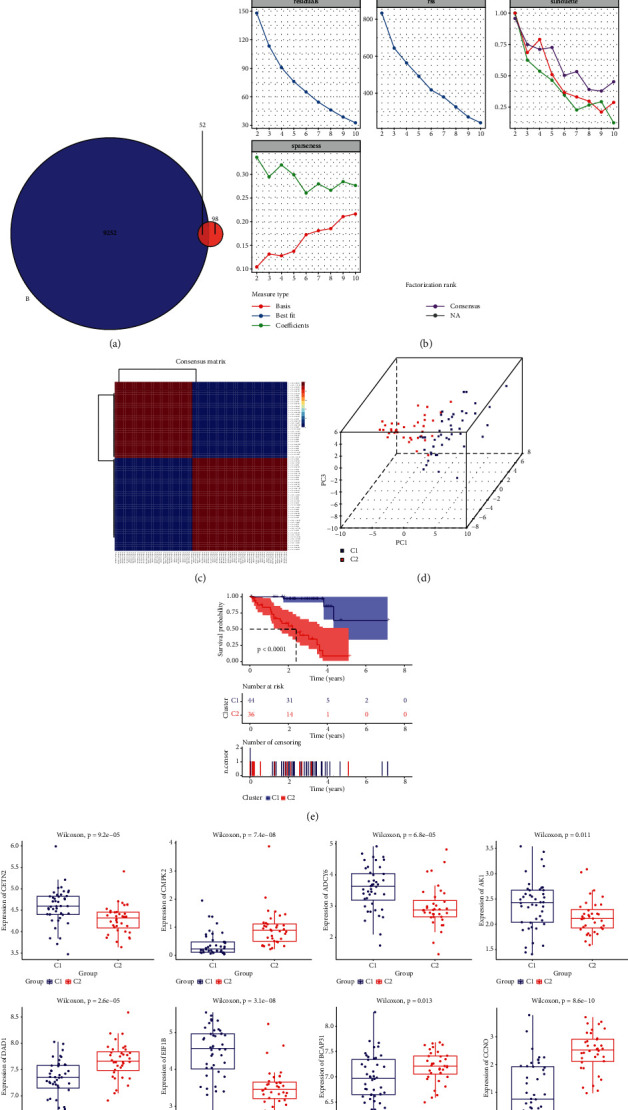
(a) Venn plot of DNA-related regulators. In total, 52 genes were revealed in the intersection between survival-associated genes and DNA repair-related genes. (b) *k* = 2 is the best value. (c) 80 UM patients were clustered by nonnegative matrix factorization (NMF) methods. (d) PCA showed a significant difference between two subtypes. (e) Survival curves for patients with two subtypes. (f) Boxplot depicting expression levels of some DNA repair genes in the C1 and C2 subtypes.

In our study, we extracted the survival information and transcriptome data of 80 UM patients from TCGA database and 150 DNA repair genes from GSEA database, and then, we performed univariate cox regression analysis on all genes and identified 9304 prognosis-related genes. Finally, 52 prognosis-related DNA repair genes were mined and selected in NMF cluster analysis (Figure 1(a)). As shown in Figure 1(b), *k* = 2 is the best value. The consensus matrix revealed cluster 1 and cluster 2, namely, C1 (*n* = 44) and C2 (*n* = 36) (Figure 1(c)). As shown in PCA plot, 80 patients were well divided into two different subtypes according to the expression pattern of 52 DNA repair genes (Figure 1(d)). Survival analysis showed that C2 subtype was a high risk subtype, and the prognosis of its patients was worse than that of C1 subtype (Figure 1(e)). As shown in Figure 1(f), DNA repair gene expression was statistically different between two subtypes.

### 3.2. GSEA of the Two Subtypes

**Figure 2:**
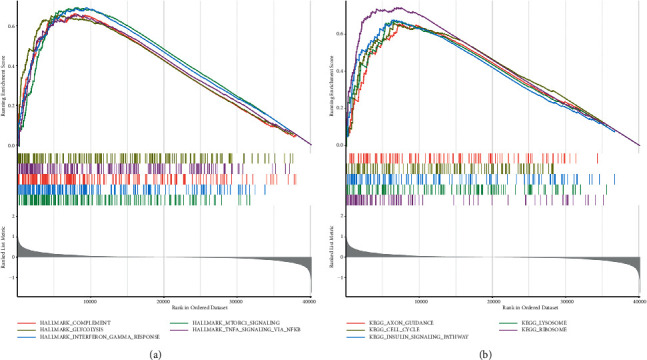
(a) Top five enrichment terms (ranked in descending order of NES). *P* < 0.05 was defined as statistically significant. (b) Top five KEGG terms.

We selected gene sets with statistical significance in the GSEA and ranked these sets according to normalized enrichment scores (NES). The top five results are displayed in Figure 2(a). We found that HALLMARK_COMPLEMENT, HALLMARK_GLYCOLYSIS, HALLMARK_INTERFERON_GAMMA_RESPONSE, HALLMARK_MTORC1_SIGNALING, and HALLMARK_ TNFA_SIGNALING_VIA_NFKB were all activated in the C2 subtype. KEGG pathway analysis showed KEGG_AXON_GUIDANCE, KEGG_CELL_CYCLE, KEGG_INSULIN_SIGNALING_PATHWAY, KEGG_LYSOSOME, and KEGG_RIBOSOME enrichment primarily in the C2 subtype (Figure 2(b)).

### 3.3. Difference in SNVs between Two Subtypes

**Figure 3:**
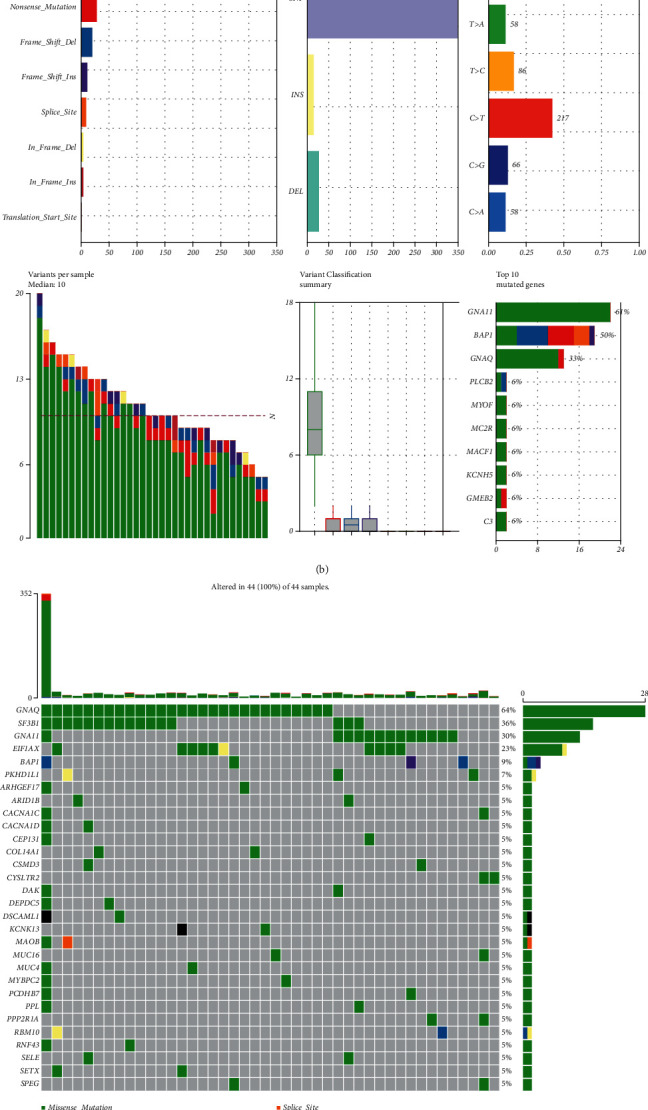
(a, b) The mutation landscape in C1 and C2 subtype. (c, d) The top 30 genes with the highest mutational frequency in C1 and C2 subtype.

Furthermore, we analyzed the mutation landscape in C1 and C2 subtype using the “maftools” R package (Figures 3(a) and 3(b)). The top 30 genes with the highest mutational frequency were selected and visualized (Figures 3(c) and 3(d)). Among the top 30 genes with the highest mutation rates in C1 and C2 subtypes, only GNAQ, BAP1, GNA11 and SF3B1 genes are the same. Compared with C1 subtype, 26 genes in C2 subtype, such as PLCB4, FBN1, EP400, AR, APC, ANXA2, ANKRD27, and ANKK1, may be associated with poor prognosis.

### 3.4. Construction of 6-Gene Risk Model

**Figure 4:**
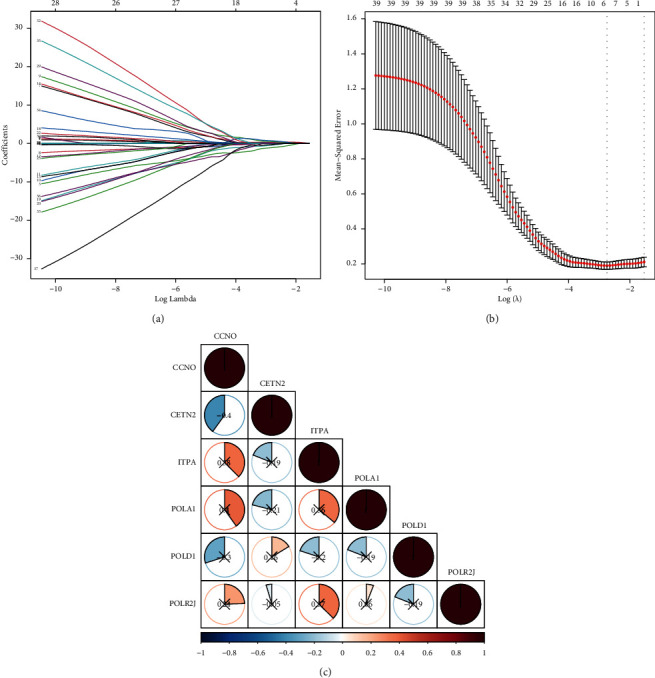
(a, b) Six key genes for prognostic value using the LASSO model. (c) The association among these six genes.

We sought to identify the key genes for prognostic value using LASSO model and ultimately found out six genes including CCNO, CETN2, ITPA, POLA1, POLD1, and POLR2J (Figures 4(a) and 4(b)). Pearson's correlation coefficient was used to examine the association among these six genes (Figure 4(c)).

### 3.5. Validation of the Model

**Figure 5:**
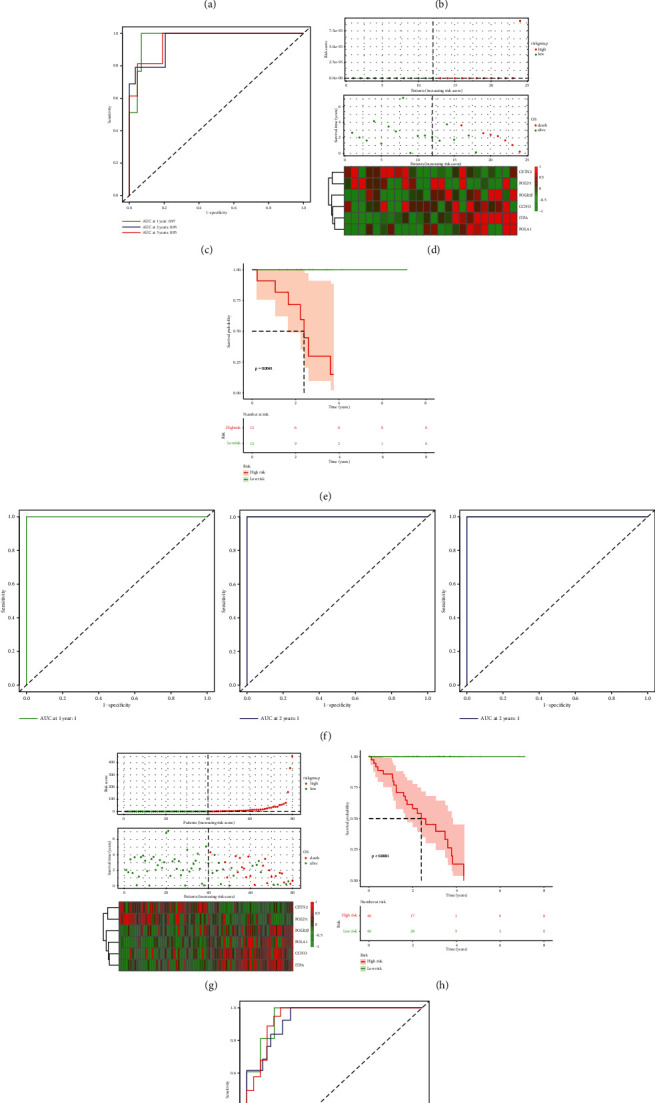
(a–i) The distributions of risk scores, alive/dead status, and expression of DNA repair genes, Kaplan–Meier curves of patients in high risk and low risk about OS, and ROC curve of 1, 2, and 3 years in the training group (a–c), in the internal validation group (d–f), and in the entire data set (g–i).

Next, patients in the training group (*n* = 56), internal validation group (*n* = 24), and entire data set were ranked according to risk scores. The expression levels of these 6 genes were evaluated in the three groups, respectively, and the stability of the results were robust. Kaplan–Meier survival curve and tdROC analyses were used to evaluate the effectiveness of the risk model in three data sets. As shown in Figures 5(a)–5(h), patients with higher risk scores had a worse OS. In the tdROC, the AUC values all exceeded 0.9, indicating a good fit (Figures 5(c), 5(f), and 5(i)).

### 3.6. Construction of Nomogram in UM Patients

**Figure 6:**
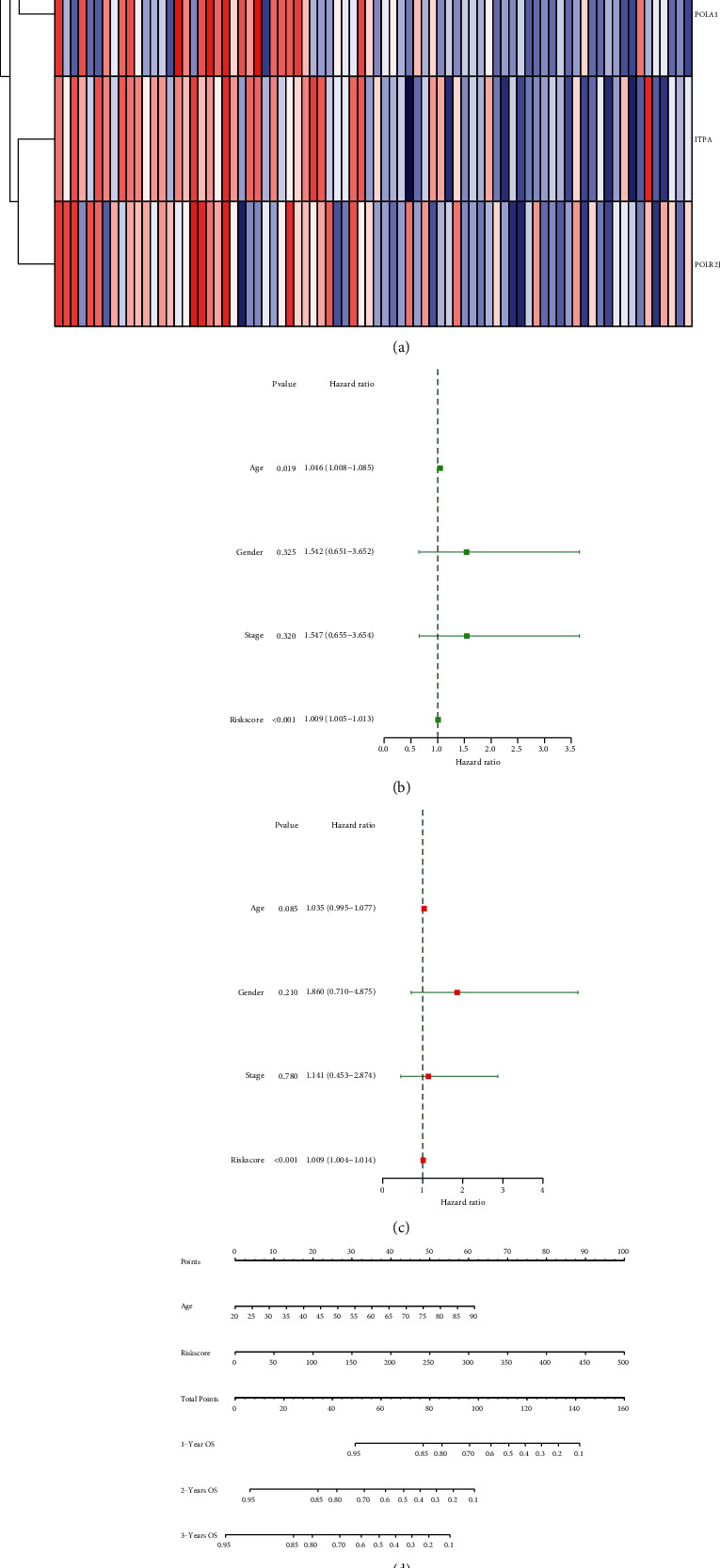
(a) The correlation between their expression and other clinical characteristic. (b, c) Univariate and multivariate cox regression analysis results. (d) The nomogram based on age and risk scores. (e) The calibration curve of 1, 2, and 3 years.

The clinical characteristics combined with the expression of six core DNA-repair genes are shown in the Figure 6(a). Then, we performed univariate and multivariate cox regression analysis on clinicopathological parameters and risk scores including age, gender, and tumor stage to analyze their predictive ability for overall survival (Figures 6(b) and 6(c)). A nomogram was constructed based on age and risk score. The 1-, 2-, and 3-year survival time of each patient was predicted according to the nomogram (Figure 6(d)). At the same time, the calibration curve is used to evaluate the prediction efficiency of the model (Figure 6(e)). The AUC of the nomogram model is 0.973, 0.956, and 0.815, respectively (Supplementary Materials (available here)).

### 3.7. Enrichment Analysis Results

**Figure 7:**
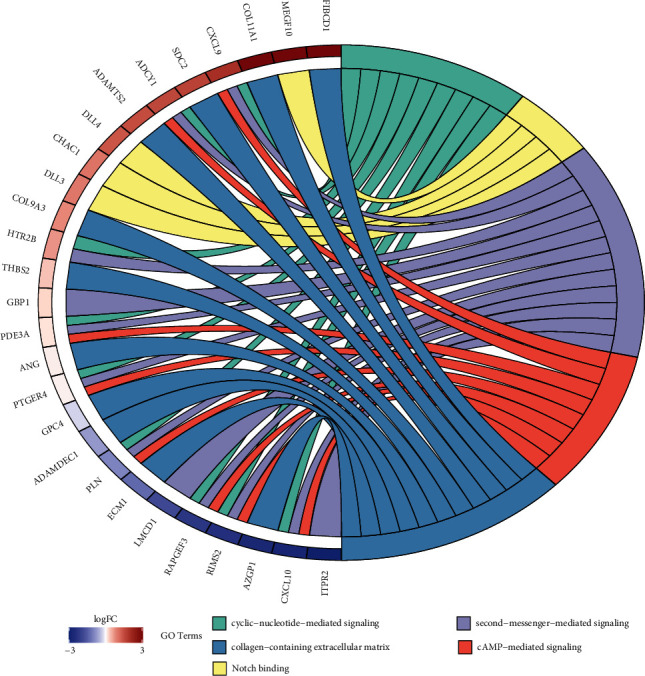
Top five GO terms.

GO enrichment analysis and KEGG pathway analysis were performed to identify the different functions and related signaling pathways between high risk and low risk groups. However, KEGG pathway analysis showed enrichment in signaling pathways has statistical insignificance. GO enrichment analysis showed that genes were enriched in cyclic-nucleotide-mediated signaling, notch binging, second-messenger-mediated signaling, cAMP-mediated signaling, and collagen-containing extracellular matrix. Compared with the low risk group, the enrichment of these functions and related signaling pathways may lead to a worse prognosis in the high risk group (Figure 7).

### 3.8. Immune Landscape of Different Groups

**Figure 8:**
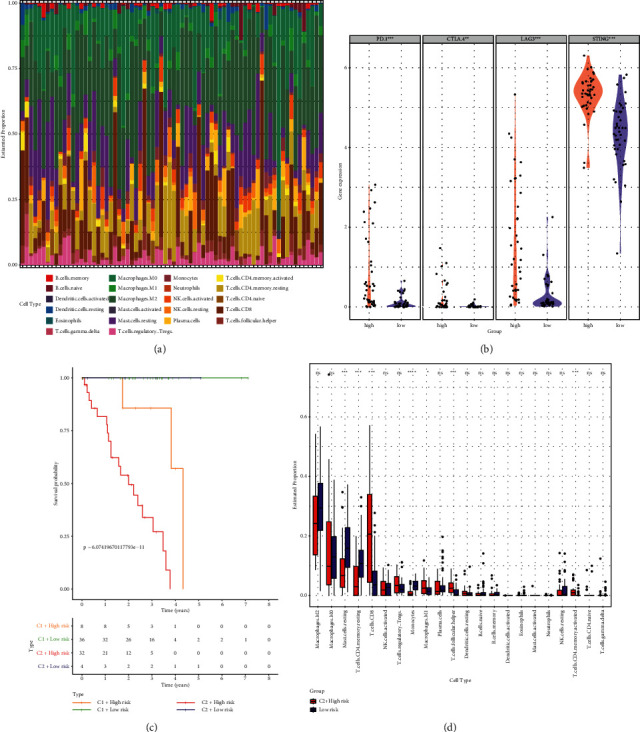
(a) The different immune cells infiltration in UM patients. (b) Expression of four common immune checkpoints. (c) Kaplan–Meier survival curve analysis. (d) The difference in enrichment levels of 22 immune cells between C2 subtype in the high risk group and low risk group.

We conducted CIBERSORT analysis to investigate the different immune cells infiltration in 80 UM patients (Figure 8(a)). We also analyzed the differential expression of four common immune checkpoint molecules including PD 1, CTLA 4, LAG 3, and STING. We found that the high risk group has a higher expression of PD 1, CTLA 4, LAG 3, and STING (Figure 8(b)). In order to determine the highest risk group, we established several new groups including the C1 subtype in the high risk group, C1 subtype in the low risk group, C2 subtype in the high risk group, and C2 subtype in the low risk group. Kaplan–Meier survival curve analysis presented that C2 subtype with high risk was the group with worst prognosis, compared with the other three groups (Figure 8(c)). Wilcox test was performed to identify the difference in enrichment levels of 22 immune cells between C2 subtype in the high risk group and low risk group. The result indicated that C2 subtype in the high risk group had the significant levels of resting mast cells, resting memory CD4 T cells, CD8 T cells, monocytes, macrophages M1 cells, follicular helper T cells, and activated memory CD4+ T cells (Figure 8(d)).

## 4. Discussion

As the most common malignant primary intraocular tumor in adults, the mortality of UM patients within 5 years is more than 95%. Because of its unique biological and clinical behavior, more than half of the patients have a poor prognosis, so it is vital to find effective prognostic biomarkers [24].

DNA repair is essential for the protection of genomes by reducing the number of mutations that may lead to carcinogenicity [25]. However, its role in UM has not been clearly understood. Here, we introduced the relationship between DNA repair genes and UM patients to identify its expression pattern and find new prognostic-related therapeutic targets.

In this study, we mined TCGA database through bioinformatics analysis, and the intersect was taken between DNA repair genes and prognosis-related genes and yielded 52 genes. Our results showed that UM patients can be divided into C1 and C2 subtypes based on the 52 genes. GSEA results indicated that abundant cancer-promoting functions and signaling pathways were activated in C2 subtype and the proportion of SNVs was higher in C2 than in C1 which suggested a worse prognosis. In the meanwhile, we built a six DNA repair genes model including ITPA, CETN2, CCNO, POLR2J, POLD1, and POLA1 by LASSO regression to predict prognosis of UM patients and utilized median value of risk scores as the cutoff point to differentiate the high risk and low risk group. The survival analyses and ROC curve in the validation group and entire data set confirmed the accuracy of this model. Inosine triphosphate pyrophosphatase (ITPA) encoded by ITPA gene in mammals can hydrolyze ITP and dITP into purine nucleoside monophosphate and pyrophosphate [26,27]. CETN2, a calcium-binding protein, is possible related to the proper duplication and segregation of the centrosome [28]. CCNO, essential for DNA damage repair, is an essential regulator of endogenous apoptosis [29]. POLR2J gene encodes submit of RNA polymerase II. Researchers have found that there may be a connection between POLR2J gene family and several hRPB11 proteins in humans [30–32]. POLD1 gene encodes the 125 kDa catalytic subunit of DNA polymerase delta, which is essential for proofreading and fidelity in DNA replication [33]. POLA1 encodes the catalytic unit of DNA polymerase *α*, which together with the primase complex launches the DNA replication process [34]. However, the role of these genes in UM patients is unclear. Survival analysis showed that the expression of these six genes was related to poor prognosis, which provided new directions for exploring the mechanism of UM occurrence and development in the future. We also constructed a nomogram based on age and risk scores to evaluate the relationship between risk scores and clinical prognosis. The calibration curve of the 1-, 2-, and 3-year OS indicated that the performance of this model is steady and robust.

Finally, the enrichment analysis showed that there were complex regulatory mechanisms in UM patients. The immune infiltration analysis indicated that the immune infiltration in C2 with the high risk group was different from that in the low risk group, which may be the reason for the worst prognosis. Previous studies have suggested that the combination of immunotherapy (IT) and radiotherapy (RT) seems to be a safe treatment, which can be divided into two categories according to the combination timing: (1) combination of RT and IT after the tumor escapes the immune system; (2) combination of RT and IT during the induction phase [35]. Our study shows that there are different immune infiltration patterns in UM patients, which may have an impact on the effect of combined treatment of IT and RT. Therefore, we can further study these different functional pathways and immune infiltration patterns, so as to improve more choices for the treatment strategies of UM in the future.

## 5. Conclusion

In this study, we identified two subtypes, C1 and C2. The C2 subtype was related to a worse prognosis. Abundant cancer-promoting functions and signaling pathways were activated in C2 subtype, and the proportion of SNVs was higher in C2 than in C1 which suggested a worse prognosis. We constructed 6-gene risk model and nomogram based on age and risk score, which can perfectly predict the prognosis of UM patients. Finally, our results showed immune cells infiltration plays essential role in prognosis.

## Figures and Tables

**Table 1:** Clinical characteristics of UM patients.

Characteristics	TCGA-UM
*Age*	
<65	45 (56.25%)
≥65	35 (43.75%)

*Gender*	
Female	35 (43.75%)
Male	45 (56.25%)

*Tumor stage*	
Stage I	0
Stage II	36 (45%)
Stage III	40 (50%)
Stage IV	4 (5%)

*Tumor diameter* (mm)	
<20	60 (75%)
≥20	19 (23.75%)
Unknown	1 (1.25%)

*Tumor thickness* (mm)	
<10	29 (36.25%)
≥10	51 (63.75%)

*Person neoplasm cancer status*	
With tumor	9 (11.25%)
Tumor-free	56 (70%)
Unknown	15 (18.75%)

*Mitotic count*	
<20	42 (52.5%)
≥20	11 (13.75%)
Unknown	27 (33.75%)

## Data Availability

The data used to support the findings of this study are available from the corresponding author upon request.

## Supplementary Materials

Supplementary Materials The AUC of the nomogram model is 0.973, 0.956, and 0.815, respectively. The AUC of (a) 1 year; (b) 2 years; (c) 3 years.
