# Supplementary figures and images for: DNA Repair Genes Are Associated with Subtype Classification, Prognosis, and Immune Infiltration in Uveal Melanoma
Source: J Oncol. 2022 Jan 19;2022:1965451. doi: 10.1155/2022/1965451 (PMC8791741; doi:10.1155/2022/1965451)

**A**


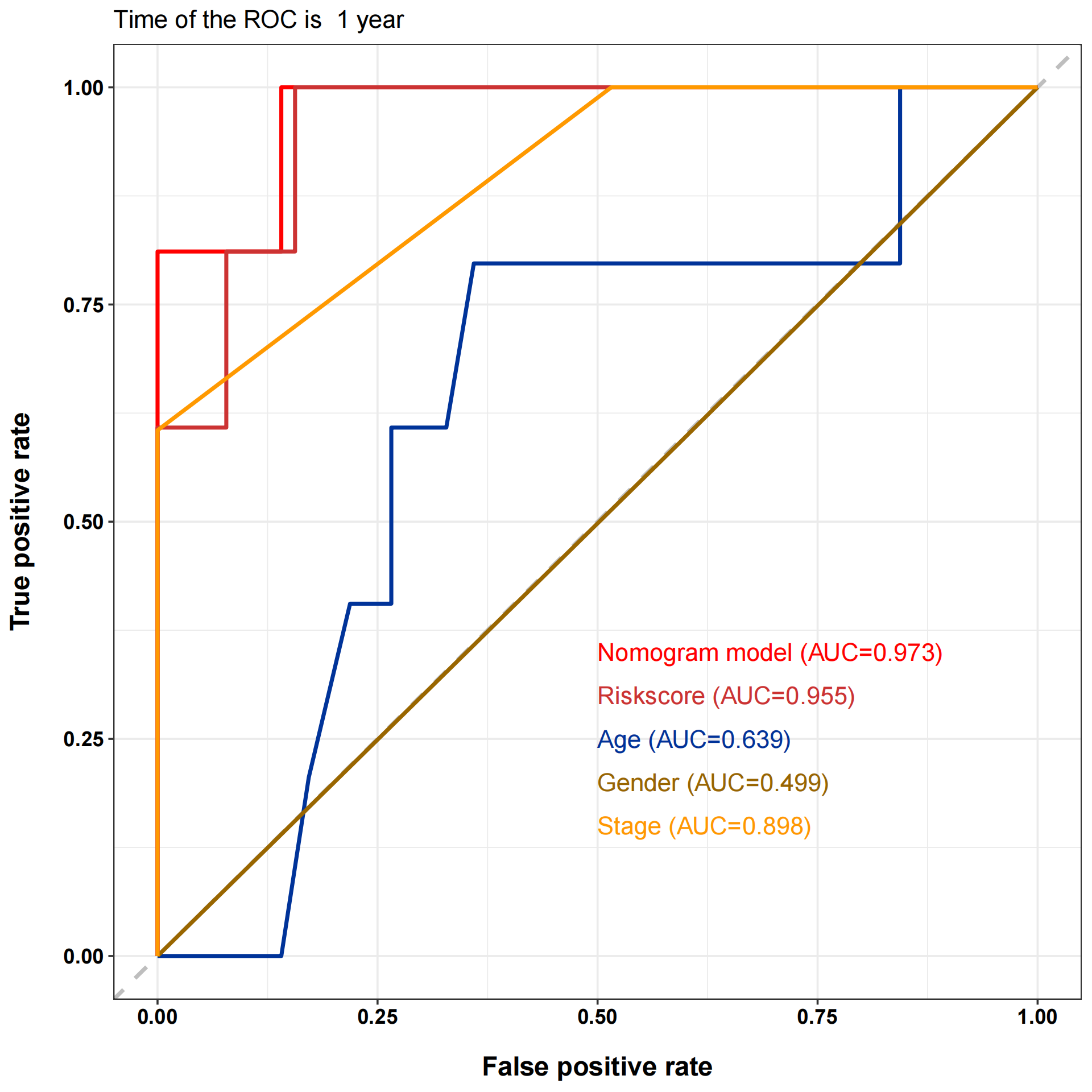


1. The AUC of 1 year

**B**


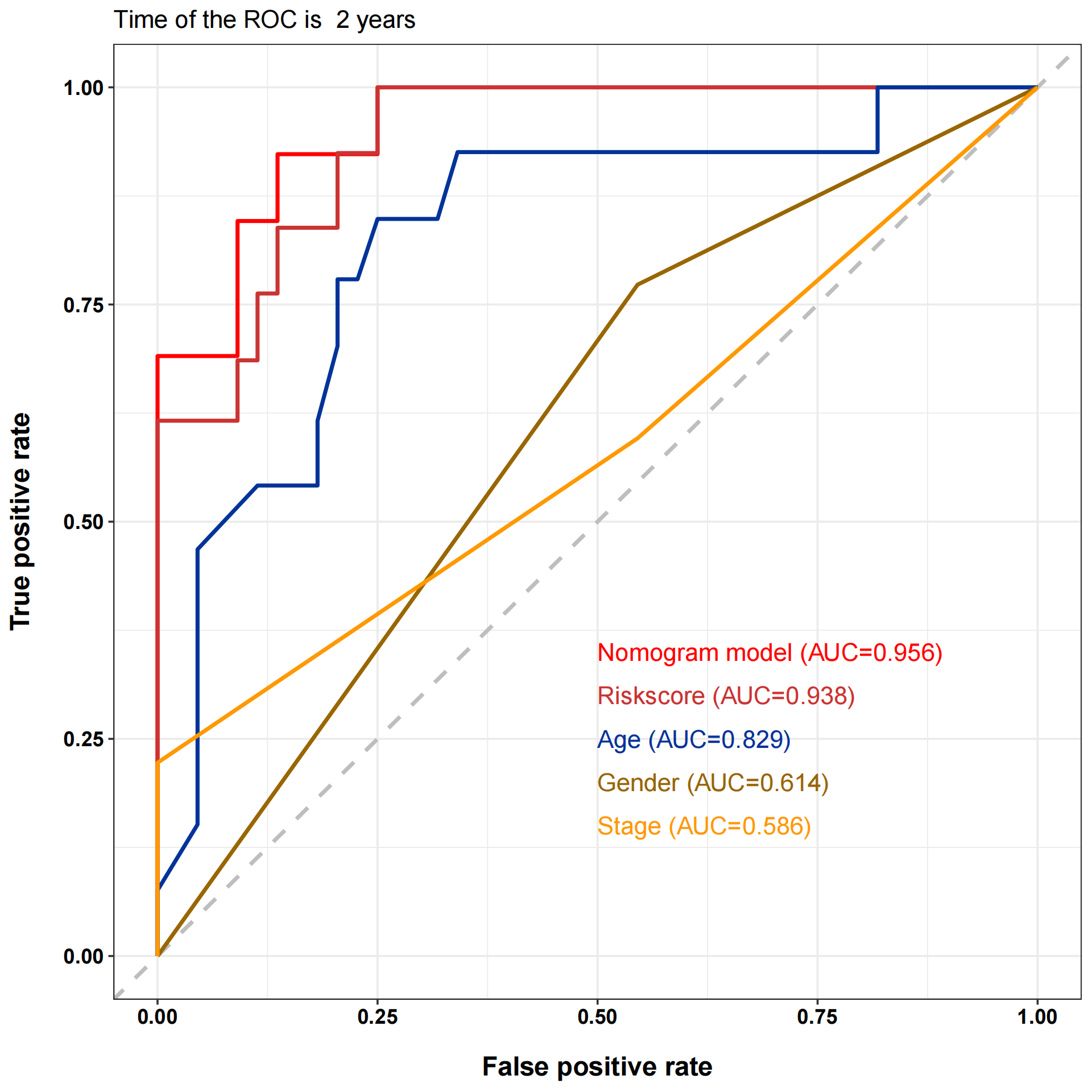


1. The AUC of 2 years

**C**


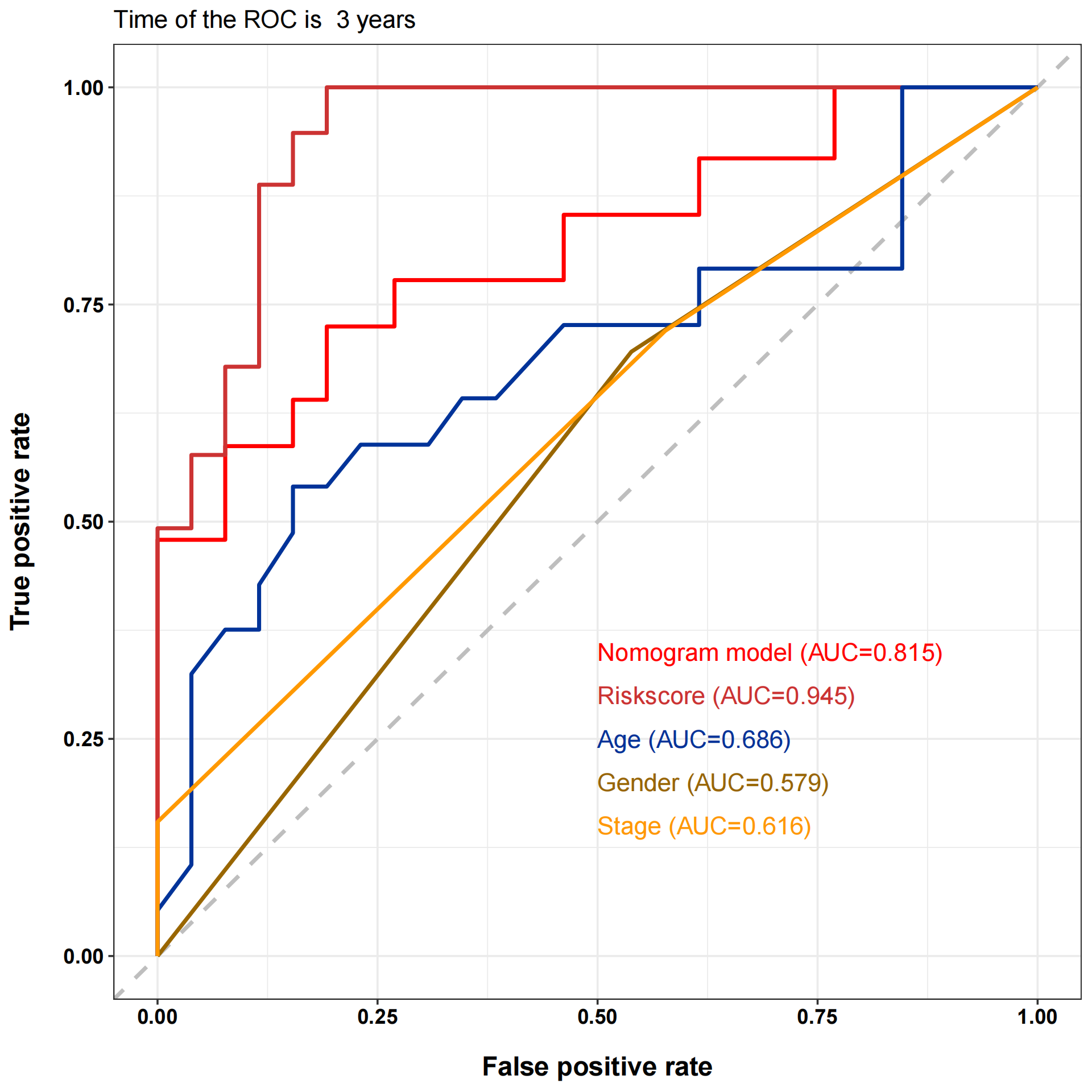


1. The AUC of 3 years

Supplement: Supplementary Materials — The AUC of the nomogram model is 0.973, 0.956, and 0.815, respectively. The AUC of (a) 1 year; (b) 2 years; (c) 3 years. [file 1965451.f1.docx]
